
JOURNAL INFORMATION
==============================
NLM Title Abbreviation: J Radiat Res
Journal ID: J. Radiat. Res
NLM Journal ID: 0376611
Journal ID: jrr

Journal of Radiation Research

ISSN: 0449-3060
EISSN: 1349-9157
Publisher: Oxford University Press

ARTICLE INFORMATION
==============================
PMCID: PMC3941497
DOI: 10.1093/jrr/rru016
Article ID: rru016
Article version: 1
Subjects: Author Index

Author Index

Print publication date: 2014 Mar
Volume: 55
Issue: Suppl 1
Issue title: Heavy Ion in Therapy and Space Radiation Symposium 2013
First page: i55
Last page: i57
Copyright: © The Author 2014. Published by Oxford University Press on behalf of The Japan Radiation Research Society and Japanese Society for Therapeutic Radiology and Oncology.
Copyright year: 2014
License: This is an Open Access article distributed under the terms of the Creative Commons Attribution License (http://creativecommons.org/licenses/by/3.0/), which permits unrestricted reuse, distribution, and reproduction in any medium, provided the original work is properly cited.
License URL: https://creativecommons.org/licenses/by/3.0/

==============================
Journal of Radiation Research, 2014, 55, i155–i157
doi: 10.1093/jrr/rru016

### Author Index

Adams, Cassandra i145
Ambrozova, Iva i141
Amemiya, N. i24
Ando, Koichi i135
Arai, H. i24
Arrizabalaga, Onetsine i55
Asaithamby, Aroumougame i83
Autsavapromporn, N. i54
Autsavapromporn, Narongchai i52
Bacikova, Alena i79, i81
Bahadori, A.A. i62
Balmain, Allan i145
Barcellos-Hoff, Mary Helen i145
Begum, Nasrin i20
Bellone, John A. i95
Benghin, Viktor i139
Bielinski, Donna i73
Bigelow, Alan i44
Boreyko, Alla i79, i81
Braby, Leslie i118
Carrihill-Knoll, Kirsty L. i100, i73
Carrozza, Joseph i111, i37
Casolino, M. i64
Casolino, Marco i139
Cengel, K. i66
Chan, R. i75
Chang, Lei i57
Chappell, L.J. i75
Chappell, Lori i50
Chen, David J. i83
Cheng, Shuk Han i113, i114, i115
Chernikch, Inna i139
Choi, Viann Wing Yan i113, i114, i115
Costes, Sylvain V. i145
Cucinotta, F. A. i12
Cucinotta, Francis A. i50
Dan, Cristian i77
Davídková, Marie i81
Davidkova, Marie i79
De Santis, Cristian i139
Demaria, Sandra i145
Desai, Sejal i116
Di Fino, L. i64
Di Fino, Luca i139
Diffenderfer, Eric S. i66, i94
Ding, Nan i57
Doetsch, Paul W. i14
Durante, Marco i55
Dynan, William S. i85
Eguchi-Kasai, K. i12
Enderling, Heiko i122
Enghardt, Wolfgang i143
Falk, Martin i79, i81
Falkova, Iva i79, i81
Fang, Yaqun i20
Fiedler, Fine i143
Fike, John i22
Ford, John i118
Frieß, Johannes i40
Fuglesang, Christer i139
Fujii, Kentaro i92
Fujimori, Akira i20, i59
Fujimoto, T. i24
Fujita, T. i24
Fukuda, Shigekazu i106, i109, i30
Funayama, T. i54
Furukawa, T. i24
Furusawa, Yoshiya i133, i135, i137, i89
Gauny, Stacey i77
Geard, Charles i44
Gee, Hannah i37
George, Kerry i50
Gerelchuluun, Ariungerel i83
Girdhani, Swati i124
Gomes, Stacey i100
Gordon, Chris i131
Goukassian, David A. i111, i122, i37
Goy, Vladimir i99
Grabham, Peter i44, i97
Grossi, Gianfranco i77
Grygoryev, Dmytro i77
Gupta, K. i42
Hada, M. i12
Hada, Megumi i50
Hartman, Richard E. i95
Hasegawa, Azusa i107
Hattori, Y. i54
He, Jinpeng i57
Hei, Tom K. i17, i70, i91
Held, Kathryn D. i32, i48
Helm, Alexander i55
Heselich, Anja i40
Higuchi, Makoto i20
Hirakawa, Hirokazu i59
Hirayama, Ryoichi i133, i135, i59
Hlatky, Lynn i124
Honikel, Louise i131
Hu, Wentao i57
Hua, Junrui i57
Hudson, Farlyn Z. i85
Idarraga-Munoz, J. i62
Ikawa, Hiroaki i107
Ikeda, H. i54
Iliakis, George i9
Illa-Bochaca, Irineu i145
Inoue, Yuko i89
Iwata, Yoshiyuki i24
Izumi-Nakajima, Nakako i20
Jakubek, J. i62
Jakubek, Jan i141
James, Gabrielle i94
Jezkova, Lucie i79, i81
Ji, Bin i20
Jones, Tamako i118
Kabarowski, J.H. i42
Kadhim, Munira A. i87
Kaisani, Aadil i46
Kamada, Tadashi i107, i26, i28
Kaminaga, Kiichi i120, i127, i129
Kanari, Yukiko i120, i127, i129
Kandimalla, Ramesh i14
Kaneko, Yumiko i133
Kartsev, Ivan S. i61
Kawamura, Hidemasa i48
Kennedy, Ann R. i66, i94
Khaled, Saman i48
Kim, Sang Bum i46
Kitamura, H. i62
Kitamura, Hisashi i70
Koba, Yusuke i109
Kobayashi, Alisa i104, i113, i114, i115, i70, i91
Kobayashi, K. i54
Kobayashi, Shingo i70
Kobayashi, Y. i54
Koda, Kana i133, i135
Kodaira, Satoshi i70
Koike, Sachiko i135
Konishi, Teruaki i104, i113, i114, i115, i70, i91
Koto, Masashi i107
Kozubek, Stanislav i79, i81
Krasavin, Evgeny A. i79
Krasavin, Evgeny A. i81
Krigsfeld, Gabriel S. i66, i94

Published by Oxford University Press on behalf of The Japan Radiation Research Society and Japanese Society for Therapeutic Radiology and Oncology 2014. This is an Open Access article distributed under the terms of the Creative Commons Attribution License (http://creativecommons.org/licenses/by/3.0/), which permits unrestricted reuse, distribution, and reproduction in any medium, provided the original work is properly cited.

Kronenberg, Amy i77
Kroupa, M. i62
Kubancak, Jan i141
Kucik, Dennis F. i42
Lai, Xianyin i131
Lamont, Clare i124
Larosa, M. i64
Larosa, Marianna i139
Larsson, Oscar i139
Laskar, S. i116
Layer, Paul G. i40
Lee, Juyong i111, i37
Li, Chunjie i48
Li, Huizi i133, i137, i59
Li, Zhentian i85
Limoli, Charles i22
Lin, L. i66
Lin, Liyong i94
Liu, Cuihua i52
Luft, Sabine i55
Lukasova, Emilie i79, i81
Lund-Jensen, Bengt i139
Lyagushin, Vladimir I. i61
Maeda, Takeshi i104, i91
Mao, Jian Hua i145
Maruyama, Katsuya i28
Maruyama, Kouichi i20
Masaoka, Aya i59
Matsumoto, Yoshitaka i133, i135, i137, i59
Michaelidesova, Anna i79
Mizota, Manabu i106
Mizushima, K. i24
Molochkov, Alexander i99
Molokanov, Alexander i141
Mori, S. i24
Morita, T. i12
Moskalyova, Svetlana I. i61
Murakami, T. i24, i54
Murakami, Takeshi i52
Murnane, John P. i85
Mutou i54
Nakajima, Mio i26
Nakajima, Tetsuo i20
Narici, L. i64
Narici, Livio i139, i18
Nasonova, Elena i55
Nelson, Gregory A. i18
Nelson, Gregory i118, i22
Nenoi, Mitsuru i20
Ng, Candy Yuen Ping i113, i114, i115
Nikolaev, Igor V. i61
Nikolaev, Viktor i139
Ninomiya, Yasuharu i20
Noda, K. i24
Noguchi, Miho i120, i127, i129, i133, i59
Nomiya, Takuma i28
Nuñez, Delia i118
Obana, T. i24
Obenaus, André i22
Ogitsu, T. i24
Oikawa, Masakazu i104, i113, i114, i115
Okayasu, Ryuichi i59, i68
Ono, Maiko i20
Ono, Tetsuya i20
Orikasa, T. i24
Ortloff, Leticia i118
Ouyang, Haoxu i145
Ozaki, Masakuni i133
Pandey, B. N. i116
Pei, Hailong i57
Peluso, Michael i124
Petrov, Vladislav M. i61
Petrov, Vladislav i139
Picozza, P. i64
Picozza, Piergiorgio i139
Pignalosa, Diana i55
Pinsky, Lawrence S. i62
Pinsky, Lawrence i141
Plante, I. i54
Ploc, Ondrej i141
Pluth, J.M. i75
Pospíšil, S. i62
Poulose, Shibu M. i73
Priegnitz, Marlen i143
Raber, Jacob i22
Rabin, Bernard M. i100, i73
Rithidech, Kanokporn Noy i131
Ritter, Sylvia i40, i55
Rohling, Heide i143
Romero-Weaver, Ana L. i66
Romero-Weaver, Ana i94
Roppel, R.D. i75
Rudobeck, Emil i102
Sakamoto, Yuka i120, i127, i129
Sanzari, Jenine K. i66, i94
Sasahara, Go i107
Sasi, Sharath P. i111, i122, i37
Sato, S. i24
Sebastiano, Christopher i145
Semones, E.J. i62
Sharma, Preety i44, i97
Shay, Jerry W. i46
Shiina, Takuya i15
Shikazono, Naoya i92
Shimazaki-Tokuyama, Yuka i89
Shinsho, Kiyomitsu i109
Shirai, T. i24
Shirai, Toshiyuki i133
Shiraishi, Iyo i92
Shirakawa, Yoshiyuki i104
Shouda, K. i24
Shukitt-Hale, Barbara i100, i73
Shurshakov, Vyacheslav A. i61
Sihver, Lembit i141, i143
Sisakyan, Hamayak i111
Smirnova, Olga Andreevna i36
Soltesz, Ivan i22
Son, H.M. i62
Song, Jin i122, i37
Sridharan, D.M. i75
Stefancikova, Lenka i79, i81
Stoffle, N.N. i62
Su, Fengtao i83
Suhara, Tetsuya i20
Sun, Maoyun i124
Suya, Noriyoshi i104
Suzuki, M. i54, i54
Suzuki, Masao i15, i52, i92
Szucs, Attila i102
Takagi, Ryo i107
Takahashi, Wataru i26
Takami, S. i24
Takayama, S. i24
Tamatsu, Satoshi i109
Tanaka, Kaoru i20
Tang, Jonathan i145
Teramura, T. i12
Terato, Hiroaki i89
Thielemann, Christiane i40
Tolochek, Raisa V. i61
Tolstonogov, Anton i99
Tsuboi, Koji i83
Tsuji, Hiroshi i107, i26, i28
Tsujii, Hirohiko i26, i28
Tungjai, Montree i131
Turecek, D. i62
Turker, Mitchell i77
Uchihori, Y. i62
Uchihori, Yukio i104, i141, i70, i91
Uehara, Yoshihiko i20
Usami, N. i54
Uzawa, Akiko i133, i135
Vachelova, Jana i79
Vlkolinský, Roman i22, i95
Vlkolinsky, Roman i102
Vykydal, Z. i62
Wakabayashi, Genichiro i109
Wan, X. Steven i66
Wang, Bing i20

Wang, Huichen i14, i85
Wang, Jufang i57
Wang, Ya i85
Wang, Yugang i91
Watanabe, Ritsuko i15
Weissman, Drew i66
Werner, Erica i14
Whalen, M.K. i75
White, C.R. i42
Wilson, Bobby L. i87
Wilson, W.C. i75
Witzmann, Frank i131
Wu, Honglu i87
Wu, Xin i57
Xu, Shuai i57
Yamamoto, Naoyoshi i26
Yamashita, Kei i133, i137
Yan, Xinhua i111, i122, i37
Yang, Gen i91
Yang, Yongyao i37
Yeshitla, Samrawit A. i87
Yokota, Y. i54
Yokoya, Akinari i120, i127, i129, i15, i92
Yoshida, K. i12
Yu, Peter Kwan Ngok i113
YU, Peter Kwan Ngok i114, i115
Yu, T. i42
Yuasa-Sunagawa, Mayumi i87
Zaconte, V. i64
Zaconte, Veronica i139
Zhang, Ye i87
Zhou, Guangming i57, i83
